# The Shape of Nasopalatine Canal as a Determining Factor in Therapeutic Approach for Orthodontic Teeth Movement—A CBCT Study

**DOI:** 10.3390/diagnostics11122345

**Published:** 2021-12-13

**Authors:** Aleksandra Arnaut, Pavle Milanovic, Milica Vasiljevic, Nemanja Jovicic, Radisa Vojinovic, Dragica Selakovic, Gvozden Rosic

**Affiliations:** 1 Department of Dentistry, Faculty of Medical Sciences, University of Kragujevac, 34000 Kragujevac, Serbia; sandra11_92@yahoo.com (A.A.); pavle11@yahoo.com (P.M.); milicavaska13@gmail.com (M.V.); 2 Department of Histology and Embryology, Faculty of Medical Sciences, University of Kragujevac, 34000 Kragujevac, Serbia; nemanjajovicic.kg@gmail.com; 3 Department of Radiology, Faculty of Medical Sciences, University of Kragujevac, 34000 Kragujevac, Serbia; rhvojinovic@gmail.com; 4 Department of Physiology, Faculty of Medical Sciences, University of Kragujevac, 34000 Kragujevac, Serbia

**Keywords:** nasopalatine canal (NPC), cone beam computed tomography (CBCT), morphometric analysis, teeth movement, maxillary central incisors (MCIs)

## Abstract

The aim of this study was to evaluate the impact of the nasopalatine canal (NPC) shape and its morphometric characteristics on expected teeth movement by assessing the distance to maxillary central incisors (MCIs) according to NPC type. The retrospective study was performed on 133 CBCT images. The following parameters were obtained: the antero-posterior diameter (A-P) of the nasal foramen (NF), canal length, A-P and mediolateral diameter (M-L) of the incisive foramen (IF), and the distance between NPC and MCIs. With the exception of being hourglass-shaped, each NPC shape showed specific impacts of NPC shape on the relationship between NPC diameters at different sections and distances to MCIs. In banana-shaped NPC, a significant correlation was observed for A-P NF diameter, while in cylindrical-shaped NPC, a significant correlation was observed for NPC length. The increase in M-L IF, A-P IF, A-P NF, and NPC length in funnel-shaped NPC may be a risk factor for interventions that could result in teeth movement. According to the results, it seems that the proposed methodological approach for analysis of CBCT slices in the anterior maxilla may offer detailed information that could be an additional tool in planning the procedures that result in expected teeth movement.

## 1. Introduction

The premaxilla, also known as the incisive bone, may have abnormal growth that could be accompanied by various malformations such as prognathism, deep bite, and protrusion [1]. The upper anterior teeth are part of the premaxilla and contribute not only to esthetics but also to physiological functions that include phonetics and mastication [2,3,4]. Maxillary incisor protrusion is considered to be one of the most frequent dental deformities [5]. There are various therapeutic approaches in the treatment of this malocclusion type [6,7,8,9]. In order to achieve desired corrections, patients with severe protrusion of anterior teeth consistently require maximum anterior teeth retraction [10]. However, the interventions that involve teeth movement must take into account the relations to other structures located in the region of the anterior maxilla, such as the nasopalatine canal (NPC).

A significant improvement in dental pretreatment protocols has been achieved in the last few decades by introducing cone beam computed tomography (CBCT). CBCT also provides accurate visualization of the spatial morphology of the NPC [11]. In addition, Uesugi and coworkers [12] reported that CBCT may be useful for simulating the post-treatment position of the maxillary incisors and NPC in order to provide safe teeth movement. On the other hand, using lateral cephalogram, the position of NPC could also be evaluated [13], although CBCT provides more detailed information about NPC such as morphology and morphometric characteristics [14].

Following the necessity to predict the impact of procedures that employ teeth movement in the anterior maxilla, Profit and Ackerman stated that maximal possible retraction of maxillary central incisors (MCIs) should be set at 7 mm [15], which is a significantly larger operating area in comparison to similar interventions in mandible due to shortage of anatomical and physiological constraints in the upper jaw [15]. The results of earlier studies confirmed that the palatal cortical plate was a limiting (anatomical) factor for MCI retractions [16,17,18,19]. However, since NPC is located between the palatal cortical plate and central incisors, it is not surprising why recent studies [20,21] have shown that their presence and morphometric characteristics should be also included as one of the key factors in planning procedures accompanied by teeth movement in the anterior maxilla in order to avoid contact of MCIs and NPC and even invasion into NPC [21]. Thus, Yu and collaborators noted that root retraction above 4 mm resulted in NPC invasion in 54% of patients [21]. Furthermore, Chung and coworkers [22] found a direct correlation between the degree of NPC invasion and severity of root resorption (the largest degree of NPC invasion presented at 6.2 mm of root resorption). Root resorption causes root shortening [23] and the consequence of this complication is manifested as tooth mobility [24]. Thus, it is not surprising that Brezniak [25] and Hartsfield [26] classified root resorption as one of the most frequent iatrogenic adverse events in orthodontic procedures, especially for maxillary incisors. Therefore, in order to avoid complications that occur after maximum incisors retraction in the anterior maxilla [27], it seems that the estimation of NPC morphometric characteristics may have an important role before orthodontic interventions. It is well known that NPC represents a long narrow structure that connects oral cavity through the incisive foramen and nasal cavity through the nasopalatine foramina [28,29,30], with previously described [31,32] content that includes the nerve, arterial terminal branches, and veins (which provide vascularization of the anterior plate between canines). It seems reasonable that accurate contact between incisors’ roots and NPC vasculature may be the pathophysiological background of root resorption.

Morphological and morphometric NPC variations are well described in the literature [33,34,35]. Moreover, the impact of NPC shapes in preoperative planning of implant placement has been confirmed in previous studies [36,37]. Furthermore, Alkanderi and coworkers [38] used virtual dental implants to explain the importance of evaluation of the distance between NPC and central incisors in order to decrease canal perforation in patients who required immediate dental implant placement. On the other hand, the role of NPC in the orthodontic interventions is insufficiently described in literature, since only a few studies [10,39] examined the morphometric relationship between NPC and central incisors.

Still, it seems that the assessment of the architecture of the region that includes both NPC and MCIs may have clinical importance in planning orthodontic interventions accompanied by significant maxillary incisors retraction [20]. Therefore, our study aimed to evaluate the potential impact of the NPC shape on expected teeth movement by means of the distance to MCIs according to NPC type.

## 2. Materials and Methods

### 2.1. Study Design

This retrospective study was based on CBCT images of patients from the Department of Dentistry of the Faculty of Medical Sciences, University of Kragujevac, Serbia, during the period from April 2018 to June 2021. The entire procedure was carried out following an approval of the institutional review board of the Faculty of Medical Sciences, University of Kragujevac (approval ID 01-4376) and in accordance with the current version of the Declaration of Helsinki. The inclusion criteria for this study were defined as follows: ≥18 years of age, presence of maxillary incisors, no history of either trauma or dental treatment related to the maxillary incisors, the absence of congenital, and/or developmental abnormalities that includes the anterior maxilla region (CBCT recordings obtained from patients that did not fulfill including criteria were excluded from this study). All selected patients were informed about the investigation protocol, and written consent to use clinical data was obtained. Following the criteria mentioned above, the total number of participants included in this study was 133 (70 male and 63 female, 45.83 ± 1.96 and 41.13 ± 1.68 average age, respectively).

### 2.2. CBCT Imaging Device and Software Characteristics

The images were obtained by using an Orthophos XG 3D device (Sirona Dental Systems GmbH, Bensheim, Germany) with three-dimensional settings for recording, VOL1 HD (85 kV/6 mA, exposure time—14.3 s) or VOL2 HD (85 kV/10 mA, exposure time—5.0 s), and a voxel size of 160 µm or 100 µm, respectively. The field of view for CBCT images was 8 × 8 cm. For the analysis of images, GALAXIS software v1.9.4 (Sirona Dental Systems GmbH, Bensheim, Germany) was used.

### 2.3. Morphometric Parameters

**Figure 1:**
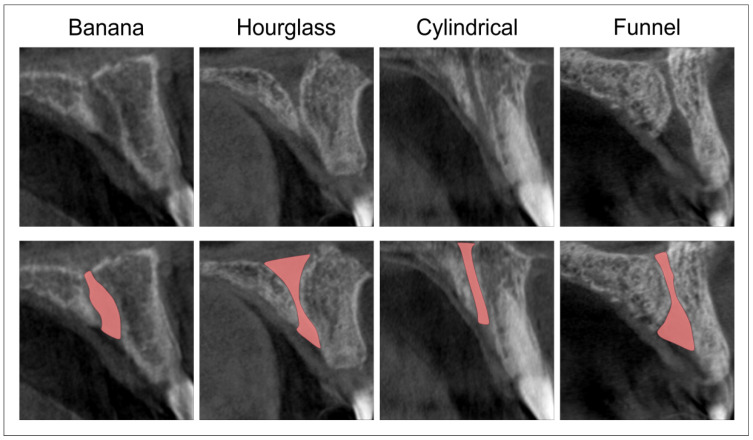
The NPC types and marks of interest. Upper: Sagittal CBCT cross-sections; bottom: red marks define NPC types.

**Figure 2:**
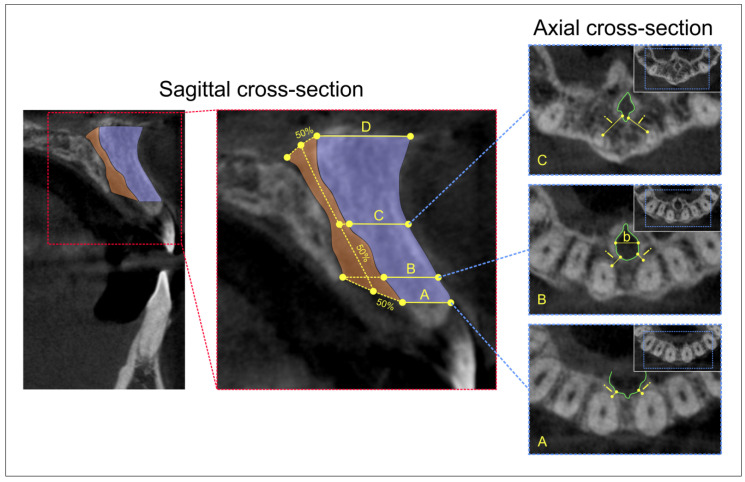
The definition of morphometric parameters of interest on CBCT images of the anterior maxilla. Sagittal cross-section; sagittal CBCT view with the marked field of interest (**left**); selected morphometric parameters for analyses (**right**): (**A**) the distance between the cortical layer of the incisive foramen and facial aspect of the buccal bone plate; (**B**) the distance between the cortical layer of the nasopalatine canal and facial aspect of the buccal bone plate using a horizontal line from the palatal border of the incisive foramen; (**C**) the distance between the cortical layer at the midpoint level of NPC length and facial aspect of the buccal bone plate; and (**D**) the distance between the cortical layer of the nasal foramen and facial aspect of the buccal bone plate. Axial cross-section; axial CBCT view: (**bottom**) the minimal interspace (i) between incisive foramen and central incisors at level A; (**middle**) the minimal interspace (i) between incisive foramen and central incisors at level B; (b) M-L diameter of incisive foramen; and (**upper**) the minimal interspace (i) between NPC and central incisors at level C.

Following the previously described criteria, we evaluated the NPC type at the sagittal view and classified them into four categories (Figure 1), as previously reported [34,40]. Moreover, by using the sagittal view, we defined four levels of relevance [37,41]—A, B, C, and D—as presented in Figure 2. Sagittal views were also used for quantification of the following diameters: the antero-posterior diameter (A-P) of the nasal foramen, canal length, and the antero-posterior diameter (A-P) of the incisive foramen. The axial view was used for the determination of the mediolateral diameter (M-L) of the incisive foramen and the distance between NPC and central incisors. It should be noted that the determination (Figure 2) of M-L diameter of incisive foramen was performed at level B, while the distance between NPC and central incisors was quantified at A, B, and C levels (due to insufficient number of images that allowed the analysis of D level, as previously addressed to anatomical variations by Vasiljevic and coworkers [37]). All parameters were analyzed by two independent observers who made the measurement blind to the protocol, with high inter-rater reliability (Pearson’s r = 0.95).

### 2.4. Statistical Analysis

All data obtained in this study were expressed (in mm) as means ± SEM. Following initial submission to Levene’s test for homogeneity of variance and to the Shapiro–Wilk test of normality, the comparisons between groups were performed using one-way ANOVA, followed by Scheffe’s post hoc test. Furthermore, Pearson’s coefficient of correlation was used to analyze relationships between parameters, and simple linear regression analyses were performed. A value of *p* < 0.05 was considered to be significant. Statistical analysis was performed with the SPSS version 20.0 statistical package (IBM SPSS Statistics 20, Armonk, NY, USA).

## 3. Results

**Figure 3:**
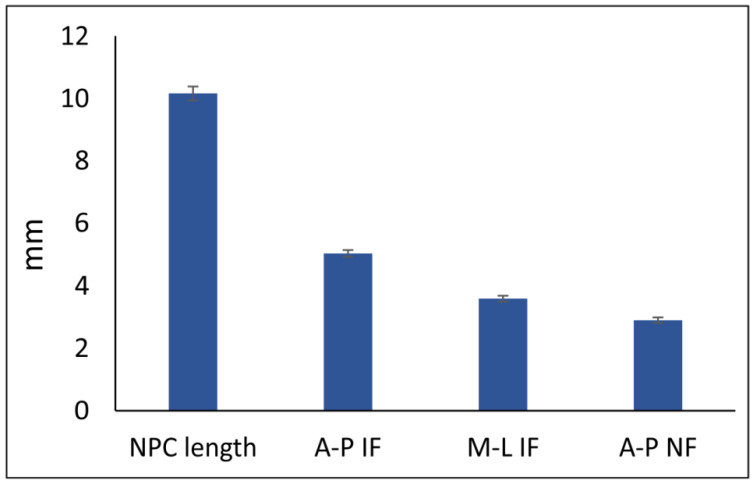
The morphometric parameters predefined for NPC.

NPC type distribution in this study was evaluated in both male (70) and female (63) patients (Table 1), and no significant gender impact on NPC type was confirmed (Pearson Chi-Square = 3.013, df = 3, *p* = 0.390). Therefore, the morphometric quantification of NPC diameters, as presented in Figure 3, included the total number of participants. 

**Figure 4:**
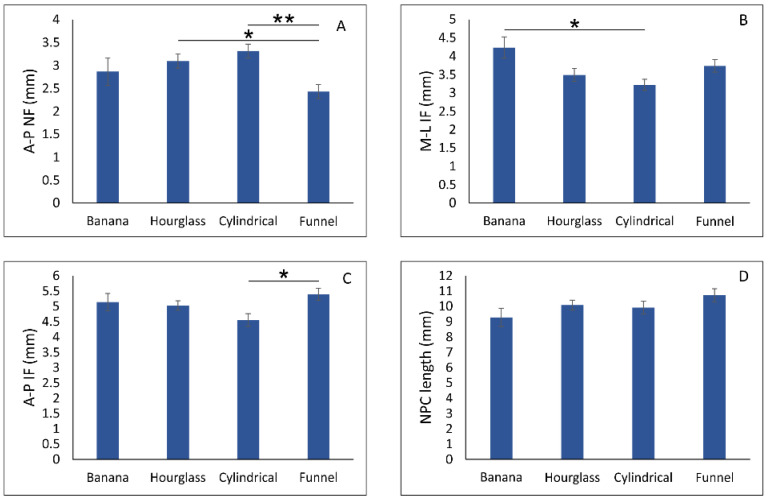
The impact of NPC type on NPC diameters at different sections. (**A**) A-P diameter of NF, (**B**) M-L diameter of IF, (**C**) A-P diameter of IF, and (**D**) NPC length. Bars represent the mean ± SEM. * denotes a significant difference of *p* < 0.05; ** denotes a significant difference of *p* < 0.01.

The evaluation of the impact of NPC type on NPC diameters at different sections (Figure 4) revealed a significant influence of NPC shape on A-P NF, A-P IF, and M-L IF (df = 3, F = 6.122, 3.512, and 3.952, respectively), with no significance for NPC length (F = 1.508). Antero-posterior diameter of the nasal foramen (Figure 4A) was significantly lower in funnel NPC type when compared to the hourglass (*p* < 0.05) and cylindrical (*p* < 0.01) NPC types. Medio-lateral diameter of the incisive foramen (Figure 4B) was significantly extended in the banana type when compared to the cylindrical NPC type (*p* < 0.05). In contrast, the A-P diameter of an incisive foramen in funnel NPC type (Figure 4C) was significantly above the values observed in the cylindrical type (*p* < 0.05). 

**Figure 5:**
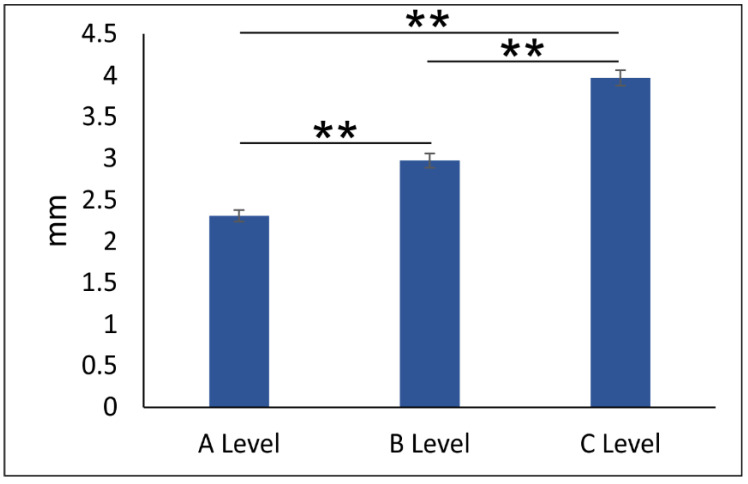
The distance between NPC and central incisors at different levels of anterior maxilla. Bars represent the mean ± SEM. ** denotes a significant difference of *p* < 0.01.

**Figure 6:**
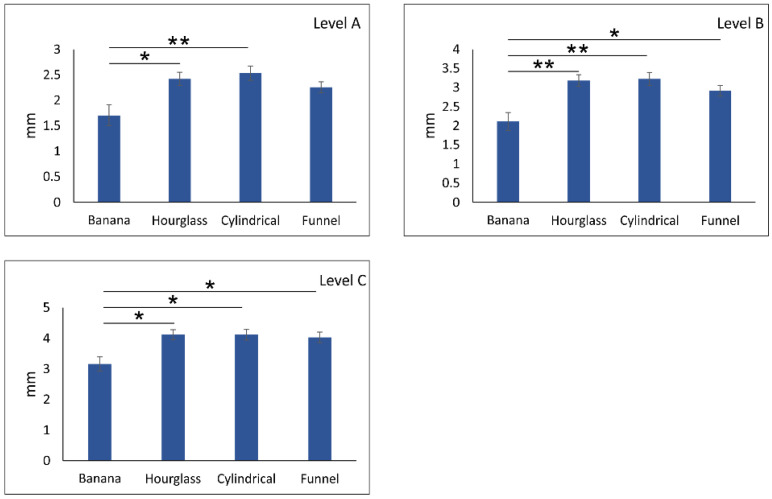
The impact of NPC type on distance between central incisors and NPC at different sections of anterior maxilla (**A**) level A, (**B**) level B, and (**C**) level C. Bars represent the mean ± SEM. * denotes a significant difference of *p* < 0.05; ** denotes a significant difference of *p* < 0.01.

As presented in Figure 5, the distance between NPC and central incisors at different levels of anterior maxilla showed a stepwise increase with significant differences among the levels (df = 2, F = 101.582, *p* < 0.01). However, the impact of NPC on the distance between central incisors and NPC at different sections of the anterior maxilla (Figure 6) was also significant for all three estimated levels—A, B, and C (df = 3, F = 4.502, 5.815, and 3.610, respectively). The impact of NPC shape on the distance between NPC and central incisors at level A was manifested by a significant reduction in distance in banana type when compared to the hourglass (*p* < 0.05) and cylindrical (*p* < 0.01) NPC types (Figure 6A). Furthermore, the distance to central incisors at level B for banana-shaped NPC was significantly reduced (Figure 6B) when compared to an hourglass and cylindrical type (*p* < 0.01) but also to funnel NPC type (*p* < 0.05). Almost the same reduction in NPC distance to the central incisors was observed in banana type when compared to other NPC shapes (Figure 6C, *p* < 0.05).

The linear regression analysis was performed in order to estimate the correlation between NPC diameter at different sections and distance to central incisors (Table 2). Interestingly, the distance between NPC and central incisors at level A was not significantly influenced by A-P NF diameter. At the same time, A-P IF and M-L IF diameters, as well as NPC length, significantly correlated to the distance between NPC and central incisors at all three predefined levels of the anterior maxilla. 

The estimation of the relationship between M-L IF diameter and distance to central incisors at different levels according to NPC type (Table 3) revealed that NPC type significantly correlated with M-L IF diameter only in funnel types at all estimated levels of the anterior maxilla. Similarly, the only significant correlation between A-P IF diameter and distance to central incisors was observed in funnel NPC type but only at level A (Table 4).

In contrast to the relationship between the incisive foramen parameters, the estimation of interconnection A-P diameter of nasal foramen and distance to central incisors depending on NPC type (Table 5), the most prominent interconnection was observed in banana type (at all three levels), while a significant correlation among other NPC types was confirmed only for funnel type at the level C.

Finally, as shown in Table 6, the analysis of the relationship between NPC length and distance to central incisors at different levels according to NPC type confirmed that in banana and hourglass NPC types, no significant interconnection was observed. At the same time, in the other two NPC types (cylindrical and funnel), a significant correlation was present at all predefined levels of the anterior maxilla (except for funnel type at the level A).

## 4. Discussion

Due to increased prevalence of malocclusion in the population [42] and possible iatrogenic trauma, such as contact between NPC and maxillary central incisors (MCIs) or NPC invasion by MCIs due to teeth movement during orthodontic treatment [10], we evaluated morphological and morphometric characteristic of NPC that could be of clinical importance in those situations. We also analyzed the relationship between NPC and MCIs by means of the impact of NPC type on the distance to MCIs at different levels.

Although using of traditional exams such as a lateral X-ray (cephalometric analyzes) may allow the identification of NPC [13], 2D imaging could not provide complete visualization of the size and position of the canal, as previously reported [14]. Thus, we classified NPC shapes by using CBCT sagittal cross section, according to Mardinger and coworkers [40], and confirmed that the most represented NPC shape was funnel (34.59%), followed by cylindrical (28.57%), and hourglass (24.81%), while the banana type was observed only in 12.03% participants. This is in line with the study by Fakuda and coworkers [33] and Lake and colleagues [43], who also reported the funnel NPC type as the most frequent shape. In contrast, the study by Gil-Marques and coworkers [34] declared the prevalence of banana shape NPC. Moreover, the results of our study showed no significant gender difference in NPC shape, which is in accordance with previous results of Milanovic [36] and Thakur and coworkers [28].

The analyses of morphometric parameters of NPC (obtained in sagittal CBCT cross-section) showed that the average NPC length observed in this study was slightly above 10 mm, which is similar to the results presented by Bronstein and colleagues [35], and was in the range between 8 mm [44] and 16 mm [45]. The average AP-IF dimension in our study (5.03 mm) is in line with the results by Kim and colleagues [46] but still above the values reported by Khojastepour and coworkers [47]. At the same time, AP-NF diameter was two-fold smaller than AP-IF, which is comparable to the results presented by Zhou and coworkers [48], and significantly below the values presented by Al-Amery and collaborators [45]. The analysis obtained in axial CBCT cross-section revealed that the average value of M-L IF diameter observed in this study (3.59 mm) was tightly fitted with Kajan [49] and Thakur and coworkers [28] (3.5 and 3.62 mm, respectively). On the other hand, Mraiwa and colleagues [44] reported that the average M-L IF diameter was 4.6 mm. The evident discrepancies in literature data considering the morphometric parameters of NPC could be addressed by the differences in methodological approach, as well as by variations in sample characteristics (gender, ethnicity, age, etc.).

Furthermore, we estimated the impact of NPC type on predefined NPC diameters (A-P NF, M-L IF, A-P IF, and NPC length). The analysis revealed that funnel NPC type was not only accompanied with significantly lower AP-NF diameter when compared to the hourglass and cylindrical type but also with a significant increase in A-P IF diameter when compared to the cylindrical shape. At the same time, significant reduction in M-L IF diameter was observed in cylindrical NPC type, while this parameter was the most prominent in banana NPC type, which is in line with previously presented observations [41]. As previously reported [41], those morphometric parameters could be of clinical relevance for the interventions in the anterior maxilla, such as implant placement. However, morphometric analyses, such as those performed in this study, could also influence other clinical aspects, since it has been shown that the increase in NPC width (M-L IF) was accompanied by higher prevalence of NPC perforation during maximum central incisors retraction [20,22]. Furthermore, the enhancement of ML-IF diameter can also be addressed for subsequent complications, such as orthodontically induced inflammatory root resorption [12,27,50]. Accordingly, since the results of this study clearly demonstrated that M-L IF dimension was influenced by NPC type and was significantly enhanced in banana NPC type, it seems that the patients with this NPC type may represent the group with a higher risk for complications during maximum central incisors retraction.

Following clinical relevance mentioned above, we also evaluated the interspace between NPC and MCIs at predefined levels [37,41]. Although a different methodological approach for this kind of analysis was proposed by Gull [10] and Cho and collaborators [39], we estimated the shortest interspace between NPC and mediopalatal surface of the central maxillary incisors roots, since the surface of the roots was confirmed in the literature as the most critical root area for touching with NPC during maxillary central incisors retraction [27,51,52]. We observed a significant difference in distance between NPC and MCIs’ roots at levels A, B, and C manifested as stepwise increase (2.30 mm, 2.97 mm, and 3.97 mm, respectively). Our results are in accordance with Matsumura and collaborators [53], who also reported shorter distances at the oral opening level of the incisive canal (3.1 mm) and increased distance at the root apex level of maxillary incisors (4.5 mm). Those results do not correspond to the observations of Gull [10] and Cho and coworkers [39], who reported that the average distance was approximately 5–6 mm, with a decrease from lower to upper levels. In addition to the obvious diversity of those data, clinical relevance of those parameters could be based on the fact that a higher rate of NPC invasion (54%) caused by incisors retraction was observed in patients where tooth movement was above 4 mm [20] than in patients with retraction below 2 mm (NPC invasion of 12%). Those data may result in a reconsideration of Profit’s and Ackerman’s recommendations for maximum root retraction of 7 mm [15], as already proposed by Ono [14]. Nevertheless, keeping with the facts, it seems argued that the potential risk for NPC invasion during maximum retraction of maxillary incisors gradually increases with decreasing distance between NPC and MCIs; thus, the highest risk was expected at the lower levels.

Additional analyses of the relationship between NPC and MCIs revealed the significant impact of NPC shape on distance between NPC and MCIs at the predefined levels. Thus, subjects with banana NPC type had lower interspace between NPC and MCIs at the levels of A, B, and C (1.70 mm, 2.11 mm, and 3.16 mm, respectively) when compared to hourglass and cylindrical NPC shapes, as well as to funnel NPC type at levels B and C. In contrast to banana NPC type, participants with cylindrical-shaped NPC showed enhanced interspace between NPC and MCIs at levels A, B, and C (2.53 mm, 3.22 mm, and 4.12 mm, respectively). Since Pan and coworkers [11] concluded that decreased interspace between NPC and MCIs significantly contributed to the contact between NPC and MCIs, it seems that the patients with banana-shaped NPC consequently expressed the highest risk for NPC invasion during maximum retraction of maxillary incisors at the lower portions of anterior maxilla.

Finally, linear regression analysis was performed to estimate the interconnection between NPC diameters at the different sections and the distance to MCIs. Our results showed significant correlation between A-P IF, M-L IF diameter, and NPC length and distance to MICs at all levels. Furthermore, we analyzed NPC diameters at different sections and the distance to MCIs according to NPC shape. With the exception of the hourglass shape, each NPC shape showed specific impact of NPC shape on the relationship between NPC diameters at the different sections and the distance to MCIs. Thus, in banana-shaped NPC a significant correlation was observed only for A-P NF diameter at levels A, B, and C (Table 5), while in cylindrical-shaped NPC, a significant correlation was observed only for NPC length at all three estimated levels (Table 6). Thus, the reduction in A-P NF diameter (at all levels) in banana-shaped NPC can be assumed as a limiting factor for tooth retraction. At the same time, the increase in NPC length in cylindrical-shaped NPC is accompanied with the higher risk for NPC invasion due to serious reduction in space needed for central incisors retraction. Finally, funnel-shaped NPC was accompanied by significant correlations for M-L IF (at levels A, B, and C), A-P IF (level A), A-P NF (level C), and NPC length (levels B and C). In conclusion, it seems that the increase in M-L IF, A-P IF, A-P NF, and NPC length in funnel-shaped NPC may be a risk factor for the interventions that could result in teeth movement. Literature data offer only the evidence that M-L diameter (NPC width) may contribute to adverse events accompanying orthodontic procedures [22,39].

## 5. Conclusions

In summarizing the results of this study, it seems that the proposed methodological approach for analysis of CBCT slices in the anterior maxilla may offer detailed information that could be an additional tool in planning the procedures that result in expected teeth movement. Thus, even brief initial insight in the observed morphometric algorithms may be employed as a checkpoint in preliminary orientation and defining exclusion criteria in order to avoid adverse events in orthodontic interventions.

## Figures and Tables

**Table 1:** NPC type distribution according to gender.

Gender (n)	The NPC Type at the Sagittal Cross-Section			
	Banana	Hourglass	Cylindrical	Funnel
Male (70)	6	20	18	26
Female (63)	10	13	20	20
Total 133	16	33	38	46

**Table 2:** Relationship between NPC diameter at different sections and distance to central incisors.

The Sections of the NPC	The Distance between NPC and Central Incisors at Different Sections		
	A Level	B Level	C Level
The A-P NF diameter	y = 0.0346x + 2.9198 R^2^ = 0.0014 *p* = 0.544	y = 0.0149x + 2.8554 R^2^ = 0.0004 *p* = 0.748	y = −0.0701x + 3.1781 R^2^ = 0.0103 *p* = 0.098
The A-P IF diameter	y = −0.2453x + 4.2626 R^2^ = 0.0546 ***p* = 1 × 10^−4^**	y = −0.2181x + 4.3448 R^2^ = 0.0652 ***p* = 2.48 × 10^−5^**	y = −0.1927x + 4.4613 R^2^ = 0.0601 ***p* = 4.67 × 10^−5^**
The M-L IF diameter	y = −0.2312x + 4.1208 R^2^ = 0.0575 ***p* = 7.79 × 10^−5^**	y = −0.1952x + 4.1674 R^2^ = 0.062 ***p* = 4 × 10^−5^**	y = −0.1908x + 4.3446 R^2^ = 0.0709 ***p* = 1.07 × 10^−5^**
The NPC length	y = −0.3037x + 10.864 R^2^ = 0.018 ***p* = 0.028**	y = −0.3788x + 11.289 R^2^ = 0.0423 ***p* = 7 × 10^−4^**	y = −0.2967x + 11.341 R^2^= 0.0310 ***p* = 0.004**

Significant correlations are bolded in colored fields.

**Table 3:** Relationship between M-L IF diameter and distance to central incisors at different levels according to NPC type.

The Relationship between the M-L IF Diameter and Distance to Central Incisors at Different Levels	The NPC Type at the Sagittal Cross-Section			
	Banana	Hourglass	Cylindrical	Funnel
The M-L IF diameter vs. A level	y = −0.0729x + 4.3593 R^2^= 0.0054 *p* = 0.688	y = −0.1858x + 3.9402 R^2^ = 0.0401 *p* = 0.107	y = 0.0579x + 3.0721 R^2^ = 0.0057 *p* = 0.517	y = −0.4948x + 4.8504 R^2^ = 0.1979 ***p* = 8.9 × 10^−6^**
The M-L IF diameter vs. B level	y = −0.0732x + 4.3896 R^2^ = 0.0072 *p* = 0.644	y = −0.1669x + 4.0222 R^2^ = 0.0454 *p* = 0.086	y = 0.0165x + 3.1654 R^2^ = 0.0007 *p* = 0.823	y = −0.3412x + 4.7299 R^2^ = 0.1552 ***p* = 1 × 10^−4^**
The M-L IF diameter vs. C level	y = −0.1638x + 4.753 R^2^ = 0.035 *p* = 0.305	y = −0.1564x + 4.1343 R^2^ = 0.0462 *p* = 0.083	y = −0.0027x + 3.2297 R^2^ = 2×10^−5^ *p* = 0.970	y = −0.2774x + 4.8527 R^2^ = 0.1583 ***p* = 9 × 10^−5^**

Significant correlations are bolded in colored fields.

**Table 4:** Relationship between A-P IF diameter and distance to central incisors at different levels according to NPC type.

The Relationship between the A-P IF Diameter and Distance to Central Incisors at Different Levels	The NPC Type at the Sagittal Cross-Section			
	Banana	Hourglass	Cylindrical	Funnel
The A-P IF diameter vs. A level	y = 0.2333x + 4.7453 R^2^= 0.0597 *p* = 0.178	y = −0.1524x + 5.3987 R^2^ = 0.0327 *p* = 0.146	y = 0.048x + 4.6779 R^2^ = 0.0021 *p* = 0.697	y = −0.3547x + 6.1968 R^2^ = 0.0791 ***p* = 6.6 × 10^−3^**
The A-P IF diameter vs. B level	y = 0.1711x + 4.782 R^2^ = 0.0422 *p* = 0.259	y = −0.0556x + 5.207 R^2^ = 0.0061 *p* = 0.533	y = −0.0353x + 4.6703 R^2^ = 0.0016 *p* = 0.728	y = −0.1137x + 5.7288 R^2^ = 0.0134 *p* = 0.272
The A-P IF diameter vs. C level	y = 0.2562x + 4.3329 R^2^ = 0.0918 *p* = 0.092	y = −0.1258x + 5.5476 R^2^ = 0.0362 *p* = 0.126	y = −0.0482x + 4.7548 R^2^ = 0.0032 *p* = 0.627	y = −0.071x + 5.6832 R^2^ = 0.0081 *p* = 0.395

Significant correlations are bolded in colored fields.

**Table 5:** Relationship between A-P NF diameter and distance to central incisors at different levels according to NPC type.

The Relationship between the A-P NF Diameter and Distance to Central Incisors at Different Levels	The NPC Type at the Sagittal Cross-Section			
	Banana	Hourglass	Cylindrical	Funnel
The A-P NF diameter vs. A level	y = 0.5014x + 2.0085 R^2^= 0.2405 ***p* = 0.004**	y = −0.0906x + 3.314 R^2^ = 0.012 *p* = 0.380	y = 0.0047x + 3.3043 R^2^ = 4 × 10^−5^ *p* = 0.958	y = −0.1531x + 2.7738 R^2^ = 0.0234 *p* = 0.145
The A-P NF diameter vs. B level	y = 0.462x + 1.8885 R^2^ = 0.2686 ***p* = 0.002**	y = −0.0411x + 3.2257 R^2^ = 0.0035 *p* = 0.638	y = −0.0393x + 3.4429 R^2^ = 0.0039 *p* = 0.592	y = −0.1346x + 2.821 R^2^ = 0.0298 *p* = 0.099
The A-P NF diameter vs. C level	y = 0.3592x + 1.7279 R^2^ = 0.1575 ***p* = 0.024**	y = −0.1267x + 3.6126 R^2^ = 0.0383 *p* = 0.115	y = −0.0872x + 3.6752 R^2^ = 0.0201 *p* = 0.222	y = −0.1511x + 3.0373 R^2^ = 0.058 ***p* = 0.021**

Significant correlations are bolded in colored fields.

**Table 6:** Relationship between NPC length and distance to central incisors at different levels according to NPC type.

The Relationship between the NPC Length Diameter and Distance to Central Incisors at Different Levels	The NPC Type at the Sagittal Cross Section			
	Banana	Hourglass	Cylindrical	Funnel
The NPC length vs. A level	y = 0.5014x + 8.4057 R^2^= 0.0651 *p* = 0.159	y = −0.261x + 10.72 R^2^ = 0.0239 *p* = 0.215	y = −0.6175x + 11.484 R^2^ = 0.0818 ***p* = 0.012**	y = −0.4838x + 11.814 R^2^ = 0.0288 *p* = 0.105
The NPC length vs. B level	y = 0.2571x + 8.7398 R^2^ = 0.0214 *p* = 0.424	y = −0.2695x + 10.946 R^2^ = 0.0357 *p* = 0.129	y = −0.6532x + 12.026 R^2^ = 0.1351 ***p* = 0.001**	y = −0.5639x + 12.368 R^2^ = 0.0646 ***p* = 0.014**
The NPC length vs. C level	y = 0.0347x + 9.1727 R^2^ = 0.0004 *p* = 0.916	y = −0.1623x + 10.756 R^2^ = 0.015 *p* = 0.327	y = −0.5355x + 12.124 R^2^ = 0.0952 ***p* = 0.007**	y = −0.3891x + 12.291 R^2^ = 0.0474 ***p* = 0.037**

Significant correlations are bolded in colored fields.

## Data Availability

Data available upon request from authors.
